# A Three-Dimensional Finite Element Analysis to Evaluate and Compare Stress Levels and Distribution Patterns in Lithium Disilicate and Zirconia Crowns

**DOI:** 10.1055/s-0046-1825876

**Published:** 2026-08-12

**Authors:** Ruchika U. Mukherjee, Shilpa S. Dandekeri, Chethan Hegde, Manoj Shetty

**Affiliations:** 1 Department of Prosthodontics and Crown & Bridge, Nitte (Deemed to be University), AB Shetty Memorial Institute of Dental Sciences (ABSMIDS), Mangaluru, Karnataka, India

**Keywords:** finite element analysis, monolithic zirconia, monolithic lithium disilicate, von Mises stress, maximum principal stress, occlusal loading

## Abstract

**Objective:**

This article aims to assess and compare the patterns of stress distribution and the biomechanical responses of monolithic zirconia and monolithic lithium disilicate crowns of different occlusal thicknesses using three-dimensional (3D) finite element analysis (FEA).

**Materials and Methods:**

A 3D FEA model of a mandibular first molar with all supporting structures was constructed. Eight models were developed to simulate clinically recommended preparation thicknesses for each material, including monolithic zirconia ( *n* = 4; occlusal reductions of 0.5, 1.0, 1.5, and 2.0 mm) and monolithic lithium disilicate ( *n* = 4; nonfunctional/functional cusp reductions ranging from 1.0/1.5 to 2.5/3.0 mm). Two loading scenarios were applied: a 225-N masticatory load at three angulations (0-degree vertical, 45-degree oblique, and 90-degree horizontal) and a 600-N axial maximum bite force. The von Mises stress (VM, used as a scalar yield indicator) and the maximum principal stress (S, used as a tensile fracture predictor for brittle ceramics) were recorded in the crown and in the underlying dentin.

**Results:**

Across all thicknesses, zirconia crowns developed higher peak internal VM stress (maximum: 456.8 MPa) but transferred less tensile stress to the underlying dentin (maximum: S ≈ 127 MPa) than lithium disilicate crowns, which transferred greater stress to the dentin (maximum: S ≈ 145.7 MPa). For both materials, non-axial loads (45 and 90 degrees) generated markedly higher stress concentrations (maximum: VM ≈ 457 MPa) than vertical loads (maximum: VM ≈ 278 MPa). Stress distribution within the lithium disilicate crown became visibly more homogeneous with increasing occlusal thickness.

**Conclusion:**

Within the limitations of this in silico model, monolithic zirconia exhibited a stress-shielding pattern in which more stress was retained within the crown and less was transmitted to the dentin, whereas lithium disilicate transmitted comparatively more stress to the dentin. Non-axial loading produced the highest stress peaks for both materials in this simulation. Because each material was modeled at the occlusal thickness clinically recommended for it, direct material-versus-material superiority cannot be inferred from these results, and clinical extrapolation should be made with caution.

## Introduction

The use of all-ceramic restorations in the posterior region has increased markedly in recent years, with clinical evidence suggesting performance comparable or superior to conventional porcelain-fused-to-metal (PFM) crowns. 1 Lithium disilicate (LDS) and zirconia CAD/CAM crowns demonstrate survival rates of 94 to 100% and 95 to 100%, respectively, over 5 to 10 years of clinical service, with relatively few technical complications. 1 Although PFM restorations were the long-standing standard, their aesthetic limitations, the risk of metal hypersensitivity, and chipping of the veneering ceramic have driven the development of monolithic, metal-free alternatives. 2

Among these alternatives, LDS and monolithic zirconia (MZ) have emerged as the two most widely used materials. 3 LDA (e.g., IPS e.max) is a hot-pressed glass-ceramic that combines high translucency with the ability to be adhesively bonded to tooth structure, 4 5 and has shown long-term clinical viability in posterior sites with reported 10-year survival rates of up to 96.7%. 6 Yttria-stabilized tetragonal zirconia polycrystal (Y-TZP) offers a flexural strength typically exceeding 1,000 MPa and high fracture toughness compared with other dental ceramics. 7 8 Bilayer zirconia restorations were prone to chipping of the veneering porcelain due to residual stresses and thermal mismatch, 9 10 which has motivated the widespread adoption of full-contour MZ. MZ crowns are highly fracture-resistant and can be prepared as thin as 0.5 mm, supporting more conservative tooth reduction. 11 12 13

The clinical choice between these two materials is not straightforward. Their elastic moduli differ substantially: zirconia is comparatively rigid (≈200 GPa, similar to metal), whereas LDS is more compliant (≈95 GPa) and closer to dentin (≈18 GPa). 3 14 This stiffness mismatch is biomechanically important: a stiffer crown may shield the underlying tooth but concentrates more stress within the restoration, whereas a more compliant crown may distribute stress more evenly within itself but transfer more stress to the cement and dentin. 15 16

Finite element analysis (FEA) is a well-established in silico tool for evaluating these complex stress states. 17 18 Recent FEA work has shown that material selection and preparation design (e.g., endocrowns vs. conventional crowns) markedly influence stress concentration in both the restoration and the abutment, 19 20 21 and that occlusal thickness is a key determinant of long-term durability and fatigue life for both materials. 22 23 Newer hybrid systems such as zirconia-reinforced lithium silicates (ZLSs) have also been investigated with FEA in an effort to combine the strength of zirconia with the bondability of glass ceramics. 24 25

Although axial loading is the most commonly studied condition, mastication and parafunction also generate substantial non-axial (oblique and horizontal) forces, which are known to be more damaging to brittle restorations. 26 27 Peak bite forces in the molar region can exceed 600 N, but it is the repetitive non-axial component of the chewing cycle that is most often implicated in fatigue failure. 28

Despite this body of work, very few studies directly compare modern MZ and LDS crowns across the full range of clinically recommended occlusal thicknesses for each material under matched axial, oblique, and horizontal loading vectors. The novel contribution of the present study is therefore to combine three variables: material type, occlusal thickness within the manufacturer-recommended range, and load angulation within a single, internally consistent three-dimensional (3D) FEA framework, in order to map how stress is distributed inside the crown and transferred to the underlying dentin under each scenario. The aim of this study was to evaluate and compare the stress-distribution patterns of MZ and monolithic LDS crowns of varying clinically recommended thicknesses under axial and non-axial simulated occlusal loading.

## Materials and Methods

### Geometry and 3D Model Construction

A solid 3D model of an intact mandibular right first molar was constructed in HyperMesh (Altair Engineering Inc., Troy, Michigan, United States) and imported into ANSYS (version 14.5; ANSYS Inc., Canonsburg, Pennsylvania, United States). Crown and root anatomical dimensions were taken from standard reference values described by Wheeler. 29 The full model included the molar (enamel, dentin, and pulp chamber), a uniform 0.25-mm periodontal ligament (PDL), 1.5 mm of cortical bone, and 3.0 mm of cancellous bone. A 100-µm uniform layer of resin cement was simulated between the prepared tooth and the crown.

### Crown Design Groups

Eight models were developed and divided into two groups corresponding to manufacturer-recommended preparation designs for each material. This design was selected deliberately to compare each material under its own clinically recommended thickness range, rather than to perform a strictly standardized material-versus-material comparison; this point is revisited in the “Limitations” section.

#### LDS Group ( *n* = 4)

- Model 1 (control): 1.5-mm non-functional cusp reduction, 2.0-mm functional cusp reduction, 1.5-mm axial/proximal reduction, with a chamfer finish line ( Fig. 1 ).
- Model 1A: 1.0-mm non-functional and 1.5-mm functional cusp reduction.
- Model 1B: 2.0-mm non-functional and 2.5-mm functional cusp reduction.
- Model 1C: 2.5-mm non-functional and 3.0-mm functional cusp reduction.

**Fig. 1:**
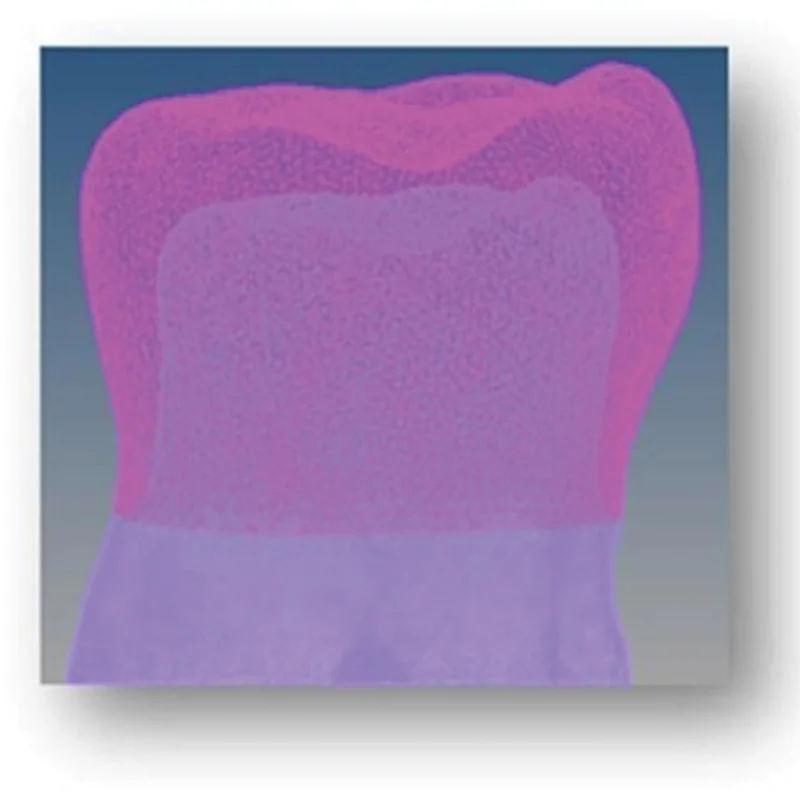
Lithium disilicate (LDS) Group, Model 1 (Control): preparation design with 1.5-mm non-functional cusp reduction, 2.0-mm functional cusp reduction, 1.5-mm axial/proximal reduction and a chamfer finish line.

#### MZ Group ( *n* = 4)

- Model 2 (control): 1.0-mm functional and non-functional cusp reduction, 1.0-mm axial/proximal reduction, 1.0-mm deep chamfer finish line with an 8-degree convergence angle ( Fig. 2 ).
- Model 2A: 0.5-mm occlusal reduction.
- Model 2B: 1.5-mm occlusal reduction.
- Model 2C: 2.0-mm occlusal reduction.

**Fig. 2:**
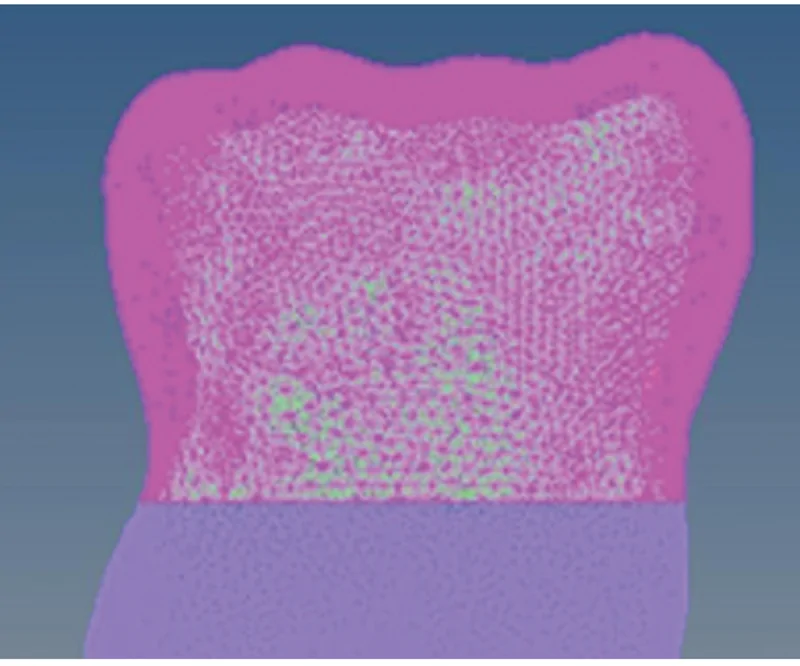
Monolithic zirconia (MZ) Group, Model 2 (Control): preparation design with 1.0-mm functional and non-functional cusp reduction, 1.0-mm axial/proximal reduction, and a 1.0-mm-deep chamfer finish line with an 8-degree convergence angle.

### Material Properties and Assumptions

All materials were modeled as homogeneous, isotropic, and linearly elastic. Young's modulus, Poisson's ratio, and density values for each component were assigned in accordance with established dental literature 3 14 and are summarized in Table 1 . Four explicit assumptions were applied throughout the model: (1) all solids were homogeneous, isotropic, and linearly elastic; (2) all interfaces (crown–cement, cement–dentin, dentin–pulp, dentin–PDL and PDL–bone) were modeled as perfectly bonded contact, with no relative slip permitted; (3) the resin cement layer was of uniform 100-µm thickness; and (4) all elements were assumed to be free of internal defects.

**Table 1:** Stress distributions on the lithium disilicate crown surface of the models loaded in three different direction simulating masticatory bite force

MODELS	Maximum Von Mises stress (MPa)			Maximum principal stress (MPa)		
	0 degrees	45 degrees	90 degrees	0 degrees	45 degrees	90 degrees
Model 1	233.059	331.874	429.699	47.182	107.65	287.512
Model 1A	246.311	321.96	424.299	50.3038	97.1999	276.59
Model 1B	241.301	315.679	393.807	39.7392	99.6491	277.208
Model 1C	262.219	305.373	414.243	53.5058	114.381	295.28

### Mesh Generation, Convergence, and Sensitivity

The geometry of each of the eight models was discretized using solid tetrahedral elements in ANSYS, which accommodate the irregular curved geometry of dental anatomical structures. A mesh-convergence study was performed prior to the main analysis: the global element edge length was progressively refined and the change in peak von Mises stress in the crown between successive refinements was monitored. The final mesh was accepted when this change fell below 5%. Local mesh refinement was applied to regions of expected stress concentration, including the occlusal contact zones, the resin–cement layer, the cervical margin, and the cusp inclines. A sensitivity check was also performed by re-running the converged mesh with a 20% variation in the assigned Young's modulus of the cement layer and of the dentin to confirm that the relative ranking of peak stresses between models was not driven by uncertainty in these specific input parameters.

### Boundary Conditions and Loading Protocol

To reproduce physiological constraint of the mandibular segment, all nodes on the mesial, distal, lingual, buccal, and inferior surfaces of the alveolar bone block were fully constrained in the X, Y, and Z directions (zero degrees of freedom).

Two loading scenarios were applied to each model:

1. **Masticatory load (225 N):** Five occlusal contact points were used, three on the inner inclines of the buccal cusps and two on the inner inclines of the lingual cusps ( Fig. 3 ) chosen to approximate the centric occlusal contacts described in classic occlusal-contact studies. 26 27 The total 225 N load was distributed equally across these five points. This load was applied in three separate simulations at 0 degrees (vertical/axial), 45 degrees (oblique), and 90 degrees (horizontal/buccolingual) to the long axis of the tooth, in order to capture the directional component of the chewing cycle.
2. **Maximum bite force (600 N, axial only):** A total of 600 N was applied vertically (0 degrees) to simulate maximum voluntary clench, distributed equally as 75 N at each of eight occlusal contact points: three on the inner slopes of the buccal cusps, two on the inner slopes of the lingual cusps, and three on the outer slopes of the buccal cusps. This larger contact-point pattern was used to reproduce the broader functional contact area reported during maximum clench, which engages additional non-working cusp slopes that are typically not loaded during gentle chewing.

**Fig. 3:**
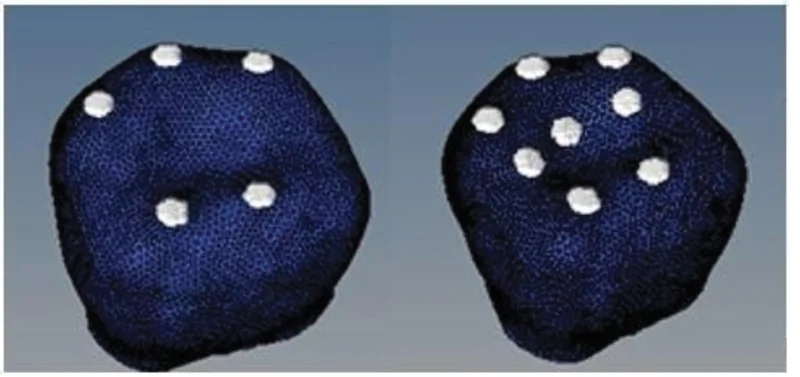
Loading locations on the occlusal surface of the mandibular first molar model. Five contact points (three buccal, two lingual; inner cusp inclines) were used for the 225-N masticatory simulations at 0, 45, and 90 degrees. Eight contact points (three inner buccal, two inner lingual, three outer buccal slopes) were used for the 600-N axial maximum-bite-force simulation.

The asymmetry in the number of contact points between the two scenarios (5 vs. 8) was therefore intentional and reflects the difference in occlusal contact distribution between light masticatory function and maximum voluntary clench rather than methodological inconsistency. The directional simulations (0, 45, and 90 degrees) were performed only at the masticatory load (225 N) because the maximum bite force at non-axial angulations would substantially exceed the physiological range reported in the literature.

### Outcome Variables and Analysis

Two stress measures were extracted from the converged solution:

- **Von Mises stress (VM):** a scalar equivalent stress, used here as an indicator of yielding for ductile components and as a global stress descriptor for ceramic restorations.
- **Maximum principal stress (S):** the maximum tensile stress, used here as a fracture predictor for brittle ceramic restorations and dentin.

Peak VM and S values were recorded at the crown and at the underlying dentin for each of the eight models under each loading condition. Qualitative stress-distribution patterns were also recorded as color-coded contour plots. No inferential statistics were performed because each FEA simulation produces a single deterministic value per condition; comparisons between models are therefore reported descriptively (higher/lower) rather than as statistically significant differences.

## Results

### Masticatory Load (225 N) Simulation

Across both materials, load angulation was the dominant determinant of peak stress in the crown. Peak VM and maximum principal (S) stresses were highest under the 90-degree horizontal load and lowest under the 0-degree vertical load. In the LDS group ( Table 2 ), the highest VM stress was 429.7 MPa in Model 1 and the highest S value was 295.3 MPa in Model 1C, both under 90-degree loading; the 0-degree vertical load produced the lowest VM stress (233.1 MPa, Model 1). In the zirconia group ( Table 3 ), the 90-degree load again produced the highest peaks (VM 456.8 MPa in Model 2 and S 300.0 MPa in Model 2A), while the 0-degree vertical load produced the lowest VM stress (229.7 MPa, Model 2C).

**Table 2:** Stress distributions on zirconia surface of the models loaded in three different direction simulating masticatory bite force

MODELS	Maximum Von Mises stress (MPa)			Maximum principal stress (MPa)		
	0 degrees	45 degrees	90 degrees	0 degrees	45 degrees	90 degrees
Model 2	256.855	338.743	456.796	56.1414	94.7552	288.54
Model 2A	278.17	346.388	433.86	98.8585	96.704	300.015
Model 2B	246.401	334.542	420.665	44.1411	101.254	270.498
Model 2C	229.732	333.611	435.603	51.1386	101.221	290.357

**Table 3:** Stress distributions on dentin surface when the models loaded in three different direction simulating masticatory bite force

Models	Maximum Von Mises stress (MPa)			Maximum principal stress (MPa)		
	0 degrees	45 degrees	90 degrees	0 degrees	45 degrees	90 degrees
Model 1	233.059	52.862	65.0419	126.772	55.4226	101.899
Model 1A	122.631	57.599	69.5208	136.848	62.0244	110.867
Model 1B	122.957	57.6738	64.4864	145.663	62.0835	100.046
Model 1C	117.235	54.6948	62.2382	126.761	56.5055	92.2555

At the dentin surface, the stress pattern was the opposite: the 0-degree vertical load was associated with the highest tensile (S) stresses. In the LDS group ( Table 4 ), the maximum S value in the dentin was 145.7 MPa (Model 1B, 0 degrees), with the 90-degree load also producing notable tensile stress (110.9 MPa, Model 1A). In the zirconia group ( Table 5 ), the maximum S value in the dentin was 127.1 MPa (Model 2C, 0 degrees). The 45-degree oblique load consistently produced the lowest VM and S stresses at the dentin surface in the zirconia group. Across the two materials, peak tensile stress in the dentin was higher under LDS (145.7 MPa) than under zirconia (127.1 MPa).

**Table 4:** Stress distributions on the dentin surface when the models were loaded in three different directions simulating masticatory bite force

Models	Maximum Von Mises stress (MPa)			Maximum principal stress (MPa)		
	0 degrees	45 degrees	90 degrees	0 degrees	45 degrees	90 degrees
Model 2	113.53	51.1133	68.9937	123.16	52.5531	111.421
Model 2A	113.297	51.213	67.1883	124.336	55.5528	105.79
Model 2B	114.971	51.652	69.5896	126.96	57.764	110.492
Model 2C	114.931	51.0738	67.3852	127.08	53.7763	107.971

**Table 5:** Maximum Von Mises stress (MPa) when masticatory force was simulated on lithium disilicate and zirconia crowns

MODELS	Lithium disilicate			Zirconia		
	0 degrees	45 degrees	90 degrees	0 degrees	45 degrees	90 degrees
Control 1 Control 2	233.059	331.874	429.699	233.059	52.862	65.0419
Model 1A Model 2A	246.311	321.96	424.299	122.631	57.599	69.5208
Model 1B Model 2B	241.301	315.679	393.807	122.957	57.6738	64.4864
Model 1C Model 2C	262.219	305.373	414.243	117.235	54.6948	62.2382

### Maximum Bite Force (600 N, Axial) Simulation

Figs. 4 to 7 show the maximum principal and von Mises stress distributions on the surfaces of the LDS and zirconia crowns under axial 600 N loading.

**Fig. 4:**
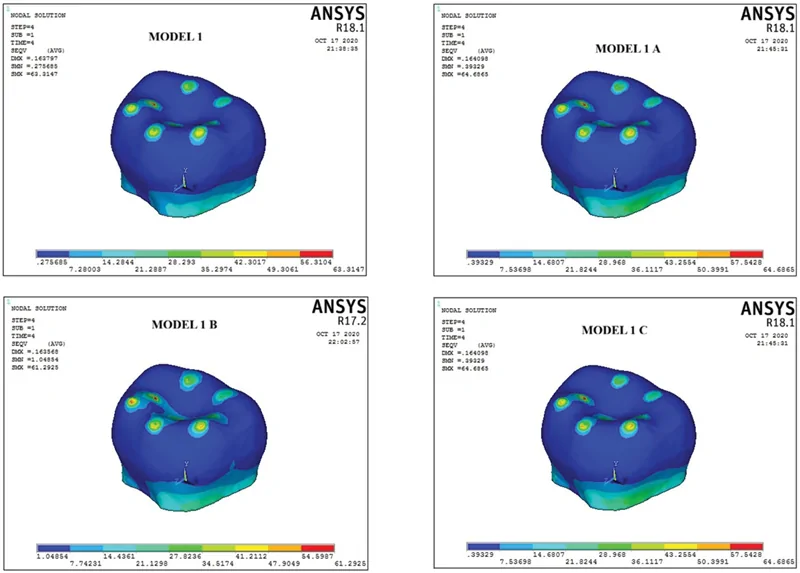
Maximum principal stress distribution on the lithium disilicate crown surface under axial 600 N loading (maximum bite force).

**Fig. 5:**
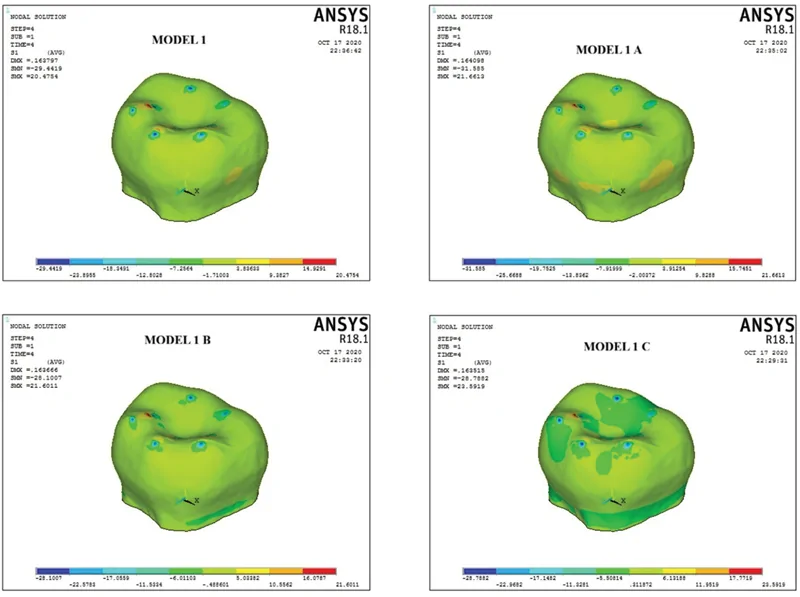
Von Mises stress distribution on the lithium disilicate crown surface under axial 600 N loading (maximum bite force).

**Fig. 6:**
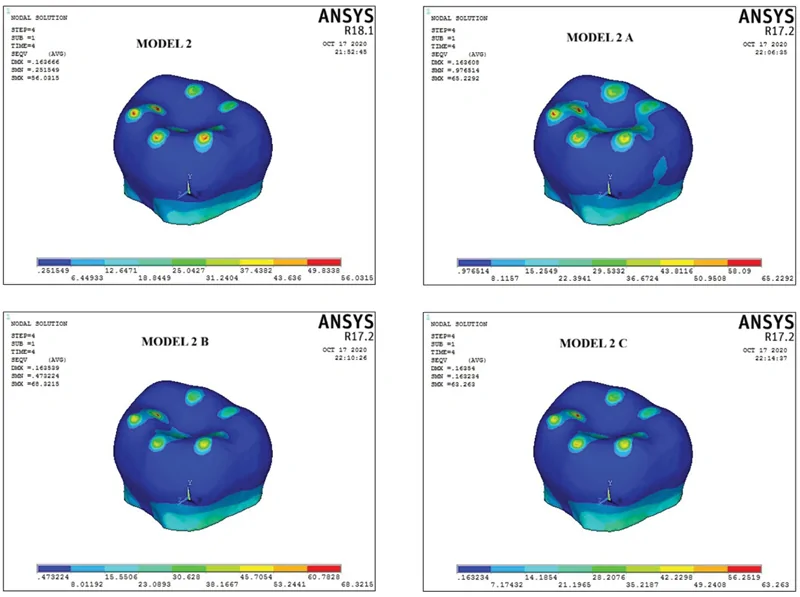
Von Mises stress distribution on the zirconia crown surface under axial 600 N loading (maximum bite force).

**Fig. 7:**
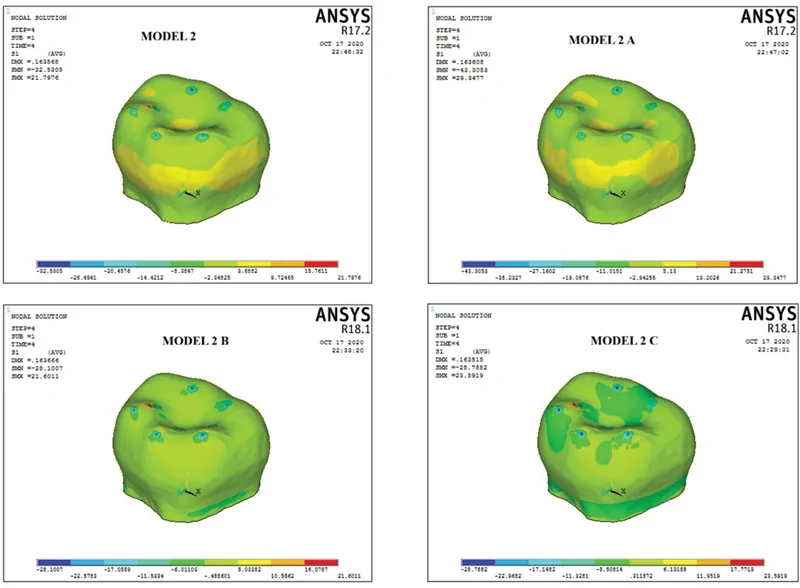
Maximum principal stress distribution on the zirconia crown surface under axial 600 N loading (maximum bite force).

In the LDS models ( Figs. 4 and 5 ), the highest principal stresses were transmitted from the occlusal loading points and propagated toward the cervical region, particularly in Models 1 and 1A. Von Mises stresses were similarly highest at the occlusal contact areas and decreased gradually toward the cervical margin.

In the zirconia models ( Figs. 6 and 7 ), peak principal stresses were predominantly localized to the occlusal surface, especially in the central fossa and at the loading points. Von Mises stresses were strongly concentrated within the occlusal portion of the zirconia crown, while comparatively lower stress was displaced toward the cervical region than in the LDS models.

Quantitatively, the peak crown stress under the axial 600 N maximum-bite-force load remained well below the values produced by non-axial masticatory loading ( Figs. 6 and 7 ). For the LDS group, peak crown VM stress ranged from 56.0 MPa (Model 1B) to 64.7 MPa (Model 1A) and peak maximum principal stress ranged from 20.5 MPa (Model 1) to 23.6 MPa (Model 1C). For the zirconia group, peak crown VM stress ranged from 61.3 MPa (Model 2) to 68.3 MPa (Model 2B) and peak maximum principal stress ranged from 20.7 MPa (Model 2B) to 29.3 MPa (Model 2A). The corresponding peak VM stresses on the dentin surface remained low for both materials (LDS: 43.3–46.1 MPa; zirconia: 42.6–43.2 MPa), reflecting the broader contact area used in the maximum-bite-force simulation, in which the load was distributed across eight contact points (including the outer buccal cusp slopes) rather than concentrated on the five inner cusp inclines used in the masticatory simulation.

## Discussion

The aim of this 3D FEA was to compare the biomechanical behavior of MZ and LDS crowns of clinically recommended occlusal thicknesses under axial and non-axial loading. The principal observations were that MZ, with its higher elastic modulus (≈200 GPa), retained more stress within the restoration and transferred less stress to the underlying dentin in the model, whereas LDS (≈95 GPa) was more compliant and transferred relatively more stress to the prepared tooth structure. For both materials, non-axial loads (45-degree oblique and 90-degree horizontal) produced markedly higher peak stress concentrations than vertical (0-degree axial) loads.

A stress-shielding pattern is a well-described characteristic of high-stiffness restorative materials. 14 15 16 In the present model, the maximum principal stress in the dentin under all zirconia configurations remained at or below 127.1 MPa (Model 2C, 0 degrees), whereas under LDS it reached 145.7 MPa (Model 1B, 0 degrees). This pattern is consistent with previous FEA comparisons of zirconia, enamel, and other ceramic systems. 18 In this simulation, the lower stress transfer to dentin was observed even at a conservative 0.5-mm occlusal preparation in the zirconia group (Model 2A), which is in line with in vitro reports of high fatigue resistance for thin MZ. 22 23 These findings should, however, be interpreted as differences in stress transfer within the model rather than as direct evidence of a clinical protective effect, since FEA does not simulate fatigue, fracture, wear, or biological response.

The greater stress transfer to dentin observed under LDS is a predictable consequence of its lower elastic modulus. 14 30 This is not necessarily an unfavorable characteristic: by deforming slightly with the underlying tooth, a more compliant crown may be loaded more uniformly. In the present analysis, increasing the LDS occlusal thickness (from Model 1 to Model 1C) produced a more homogeneous stress distribution within the crown itself ( Figs. 4 and 5 ), with reduced concentration at the cervical margin. This supports the importance of respecting the manufacturer-recommended occlusal thickness for LDS as a means of reducing localized stress peaks that have been associated with fracture in in vitro fatigue studies. 31 32 33

The strongest and most consistent finding of the present model is the marked effect of load direction. For the zirconia control crown (Model 2), the peak VM stress increased by approximately 78% between the 0-degree axial load (256.9 MPa) and the 90-degree horizontal load (456.8 MPa). A comparable trend was observed in LDS (Model 1), with an approximately 84% increase from 233.1 MPa (0 degrees) to 429.7 MPa (90 degrees). This is consistent with both clinical experience and earlier FEA studies of complex restorations such as endocrowns and cantilever designs, which have repeatedly identified oblique and horizontal forces as the most damaging. 20 34

An apparently counterintuitive observation in the present results is that, while non-axial loads produced the highest stress within the crown, the 0-degree vertical load produced the highest tensile stress in the underlying dentin. A plausible explanation is that under purely axial loading, the crown deforms primarily in compression and transmits a more focused vertical force component through the cement layer to the dentin beneath the loading points; under oblique and horizontal loading, the same force is partially absorbed and redistributed within the crown itself (and partially as shear at the crown–cement interface), so that less of the load reaches the dentin as direct tensile stress. This interpretation is consistent with the higher in-crown VM values seen at 90 degrees, and should be considered alongside the limitations of a perfectly bonded, linearly elastic model.

Two clinical implications can be drawn cautiously from the present model. First, the stress-shielding pattern observed for zirconia in this simulation may be relevant when selecting material for posterior restorations in patients requiring a more conservative (thinner) preparation, although clinical decision-making should incorporate fracture, wear, and antagonist-tooth considerations that are not captured by FEA. Second, the absolute values of stress generated under non-axial loading (429.7 MPa for LDS, 456.8 MPa for MZ) are substantial. Although these values remain well below the >1,000 MPa flexural strength reported for zirconia, the 429.7 MPa value for the LDS control crown approaches the reported biaxial flexural strength of LDS (≈400–500 MPa). 31 This reinforces the clinical importance of careful occlusal adjustment to eliminate horizontal and angular interferences in all-ceramic posterior restorations. 1

A further methodological point that warrants explicit discussion is that LDS and zirconia were modeled at the preparation thickness clinically recommended for each material, rather than at a single standardized thickness. Because thickness itself is a major determinant of stress distribution in FEA, some of the differences observed between the two material groups will reflect a combination of material properties and preparation geometry. The conclusions drawn here should therefore be limited to the comparison of each material at its clinically realistic preparation, and not interpreted as a controlled, geometry-matched material comparison.

### Limitations

Several limitations of this study should be acknowledged. The FEA model is based on a linear-static analysis with the assumption that all materials are homogeneous, isotropic, and linearly elastic 35 ; real dental materials and biological tissues are anisotropic and viscoelastic. The model did not simulate dynamic-cyclic mastication, the viscoelastic behavior of the PDL, 10 27 or thermal stresses arising from temperature changes in the oral cavity. 10 All interfaces were modeled as perfectly bonded; in clinical practice, marginal fit and adhesion vary with the material and fabrication system (pressing vs. CAD/CAM vs. additive manufacturing). 36 37 38 In silico models also do not capture material ageing, low-temperature degradation of zirconia, moisture effects or fatigue, all of which influence long-term clinical fracture and wear behaviour. 39 40 Finally, as noted earlier, LDS and zirconia were not modeled at identical occlusal thicknesses, which limits direct material-versus-material comparison.

### Future Directions

Future studies should extend this framework to dynamic-cyclic loading with viscoelastic material properties to better approximate the intra-oral environment. 10 Validation of FEA findings against in vitro fractographic analysis would strengthen the link between simulated stress patterns and observed failure modes. 41 The same loading protocol could also be applied to newer materials, including translucent 5Y-TZP zirconia, 22 42 ZLSs, 24 25 and 3D-printed ceramic composites, 43 to provide further data of clinical relevance.

## Conclusion

Within the limitations of this in silico 3D FEA of clinically recommended preparation designs, the following observations can be made:

1. MZ crowns (≈200 GPa) retained more stress within the restoration and transmitted less tensile stress to the underlying dentin than LDS crowns in this model.
2. Monolithic LDS crowns (≈95 GPa) were more compliant and transmitted comparatively higher tensile stress to the dentin (peak ≈ 146 MPa).
3. The stress distribution within the LDS crown became more homogeneous as occlusal thickness increased.
4. Non-axial (oblique and horizontal) loading was the most influential biomechanical variable for both materials, increasing peak crown stress by approximately 78 to 84% relative to axial loading and producing peak stresses that approach the reported flexural strength of LDS.

Because the two materials were modeled at different (clinically recommended) preparation thicknesses, and because FEA does not simulate fatigue, fracture, wear, or clinical outcome, these findings describe stress-distribution patterns under defined loading conditions rather than direct evidence of clinical superiority of one material over the other.
